# Supplementary material for: Mating harassment may boost the effectiveness of the sterile insect technique for Aedes mosquitoes
Source: Nat Commun. 2024 Mar 4;15:1980. doi: 10.1038/s41467-024-46268-x (PMC10912119; doi:10.1038/s41467-024-46268-x)
Supplement: Supplementary file 1 — Supplementary Information [file 41467_2024_46268_MOESM1_ESM.pdf]

Supplementary Information (SI)

Supplementary Discussion

Female mosquitoes are compulsory blood feeders and hence, the pathogen-transmitting sex. Even when irradiated, female mosquitoes require regular blood meals after release and may therefore still contribute to the transmission of diseases despite being sterile[1]. This can only be avoided if accurate sex-separating systems that remove all female mosquitoes from the release batches are available[2]. Different sexing techniques based on biological, genetic and transgenic approaches have been proposed for some mosquito species considered for SIT[3,4]. While most contemporary SIT programmes use mechanical devices to sex pupae, female contamination rates close to 1%, a threshold considered as the maximum acceptable contamination rate for release, are common[5,6]. The sex separation of *Aedes* mosquitoes is then carried out at the pupal stage, i.e., by using standard metal sieves with a square-opening mesh through which male *Aedes* swim upward, or by using the glass plate sex separation system. Given the substantial number of mosquitoes required for SIT, such methods are time-costly and require dedicated personnel to manually operate the sorting devices[4]. More recently, a sex-sorting pipeline including a mechanical pupal sieve, real-time adult visual inspection, a cloud-based machine learning classifier, and non-expert review has been described, but its cost-effectiveness remains uncertain[7,8].

When a predetermined threshold is agreed with the public health authorities, e.g., 1%[5], keeping the sterile males for 8 days might be an effective way of eliminating females instead of removing residual females manually or discarding the full batch of sterile males. Nevertheless, this would probably be cost-prohibitive in an operational programme. The feasibility of such action would require for instance, evaluating how long sterile males can be kept in the rearing facility without reducing their competitiveness. On La Réunion island, the competitiveness index of sterile male *Ae. albopictus* in semi-field conditions increased with the age of sterile males, from 0.14 one day after emergence to 0.53 after 5 days[9]. A similar result was observed in Mauritius[10] but this would require field validation.

Supplementary Figures

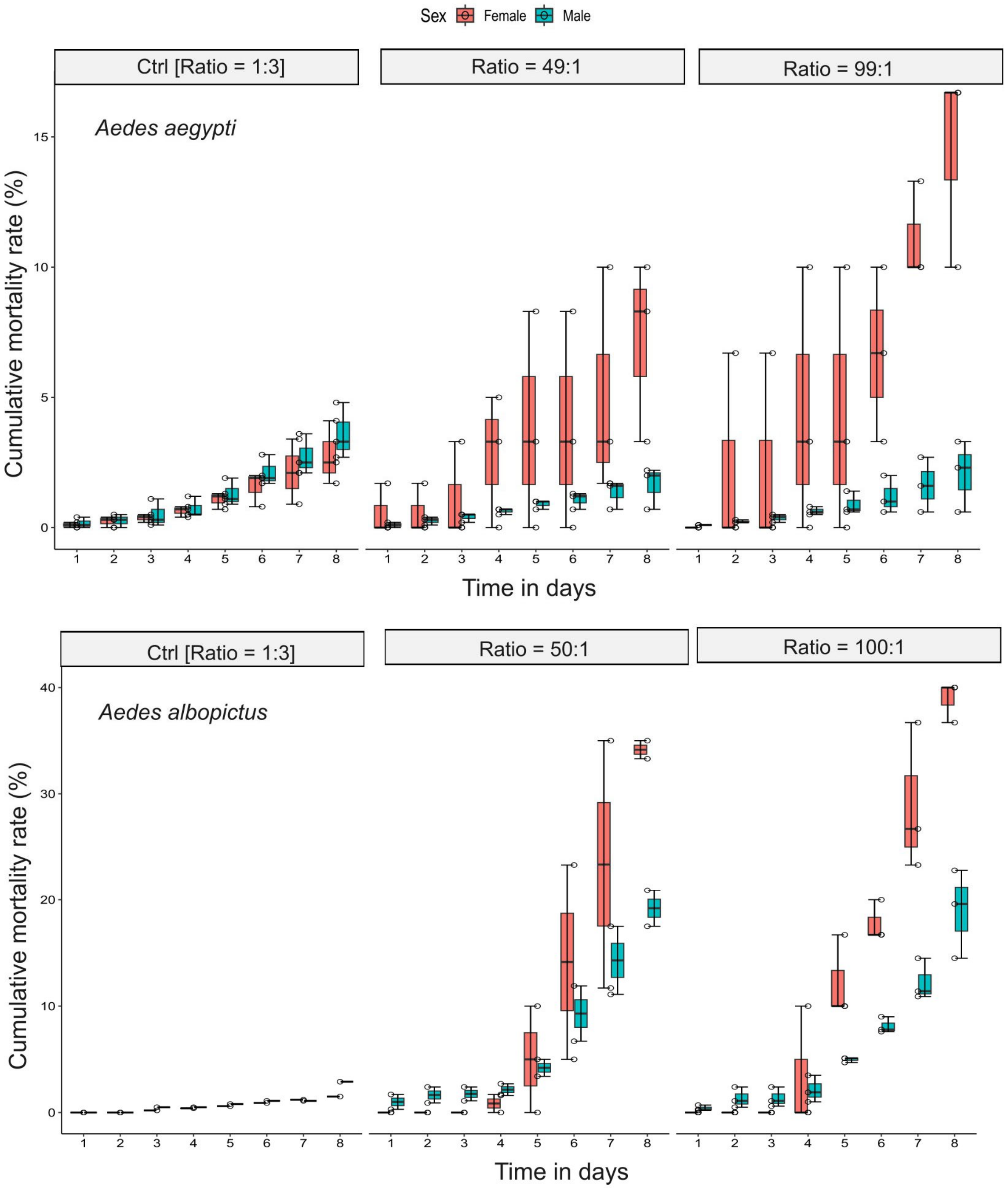

Supplementary Figure 1. Cumulative mortality rate of non-irradiated *Aedes* mosquitoes exposed to three sex ratios over 8 days. The box plots present median values and quartiles, whiskers the 95% percentiles and dots the individual data points. One-tailed pairwise multiple comparisons were performed (P value adjustment with Tukey method) using the function *emmeans ()* of the emmeans package to investigate the significance of the increase in cumulative mortality rate at different sex ratios as compared to the control (Ctrl). In *Aedes aegypti*, mortality increased in females at a sex ratio of 49:1 (odds ratio = 0.321, SE = 0.076, *z.ratio* = -4.787, $P < 10^{-4}$) and 99:1 (odds ratio = 0.166, SE = 0.037, *z.ratio* = -8.042, $P < 10^{-4}$), but not in males (at a sex ratio 49:1, odds ratio = 1.788, SE = 0.529, *z.ratio* = 1.963, *P* = 0.121

and at a sex ratio 99:1, odds ratio = 1.531, SE = 0.432, *z.ratio* = 1.508, $P = 0.286$). Treatment and control groups had $n = 3$ biologically independent replicates. In *Aedes albopictus*, mortality increased at a ratio of 50:1 both in females (odds ratio = 0.073, SE = 0.033, *z.ratio* = -5.665, $P < 10^{-4}$) and males (odds ratio = 0.104, SE = 0.044, *z.ratio* = -5.358, $P < 10^{-4}$), and also at a sex ratio of 100:1 both in females (odds ratio = 0.054, SE = 0.025, *z.ratio* = -6.339, $P < 10^{-4}$) and males (odds ratio = 0.115, SE = 0.048, *z.ratio* = -5.169, $P < 10^{-4}$). The number of biologically independent replicates was $n = 1$ for the sex ratio 1:3 (control), $n = 2$ for ratio 50:1 and n = 3 for ratio 100:1. Source data are provided in the Source Data file named "raw_data_lab&semi-field.xlsx".

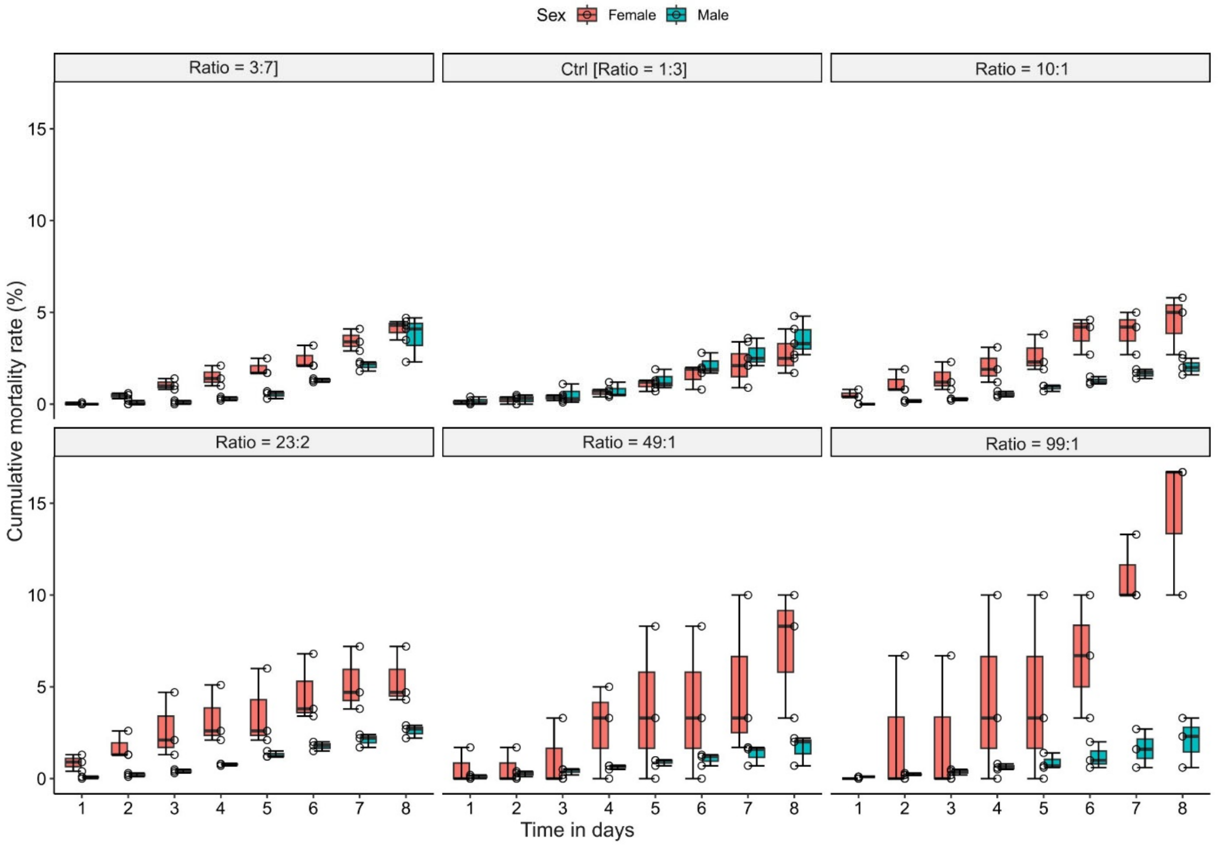

Supplementary Figure 2. Cumulative mortality rate of non-irradiated *Aedes aegypti* exposed to six sex ratios (SR) over 8 days during preliminary trials. The box plots present median values and quartiles, whiskers the 95% percentiles and dots the individual data points. One-tailed pairwise multiple comparisons were performed (P value adjustment with Tukey method) using the function *emmeans ()* of the emmeans package to investigate the significance of the increase in cumulative mortality rate at different sex ratios as compared to the control (Ctrl). No difference in female mortality was observed at a sex ratio of 3:7 (odds ratio = 0.553, SE = 0.142, *z.ratio* = -2.306, P = 0.191), but mortality increased significantly at a sex ratio of 10:1 (odds ratio = 0.387, SE = 0.094, *z.ratio* = -3.910, P = 0.001), 23:2 (odds ratio = 0.285, SE = 0.066, *z.ratio* = -5.361, $P < 10^{-4}$), 49:1 (odds ratio = 0.322, SE = 0.076, *z.ratio* = -4.782, $P < 10^{-4}$) and 99:1 (odds ratio = 0.167, SE = 0.037, *z.ratio* = -8.028, $P < 10^{-4}$) In males, there was no significant difference observed atthe sex ratio 3:7 (odds ratio = 1.461, SE = 0.407, *z.ratio* = 1.361, P = 0.750), 10:1 (odds ratio = 1.694, SE = 0.493, *z.ratio* = 1.810, P = 0.459), 23:2 (odds ratio = 1.188, SE = 0.312, *z.ratio* = 0.654, P = 0.986), 49:1 (odds ratio = 1.788, SE = 0.529, *z.ratio* = 1.963, P = 0.363) nor 99:1 (odds ratio = 1.531, SE = 0.432, *z.ratio* = 1.508, P = 0.658). Mortality rate of female reached 14.5% (SD=3.9%) after 8 days in the 99:1 batch in comparison to 2.8% (SD=1.2%) in the 1:3 control group. All treatment and control groups had n = 3 biologically independent replicates. Source data are provided in the Source Data file named “raw_data_lab&semi-field.xlsx”.

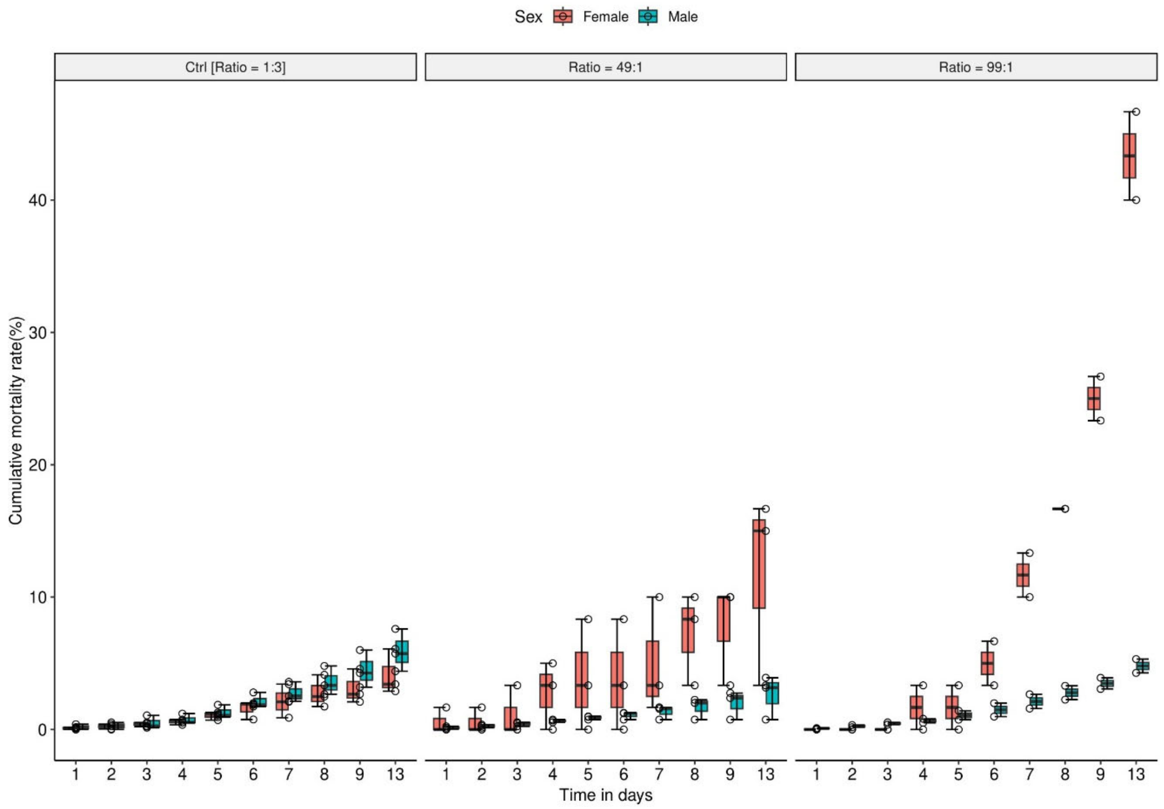

Supplementary Figure 3. Cumulative mortality rate of non-irradiated *Aedes aegypti* exposed to three sex ratios over 13 days. The box plots present median values and quartiles, whiskers the 95% percentiles and dots the individual data points. One-tailed pairwise multiple comparisons were performed (P value adjustment with Tukey method) using the function *emmeans ()* of the emmeans package to investigate the significance of the increase in cumulative mortality rate at different sex ratios as compared to the control (Ctrl). The mortality increased in females at a sex ratio of 49:1 (odds ratio = 0.348, SE = 0.0598, *z.ratio* = -6.145, $P < 10^{-4}$) and 99:1 (odds ratio = 0.107, SE = 0.019, *z.ratio* = -12.299, $P < 10^{-4}$). The number of biologically independent replicates was $n = 3$ for the sex ratios 1:3 (control) and 49:1 and $n = 2$ for ratio 99:1. Source data are provided in the Source Data file named “raw_data_lab&semi-field.xlsx”.

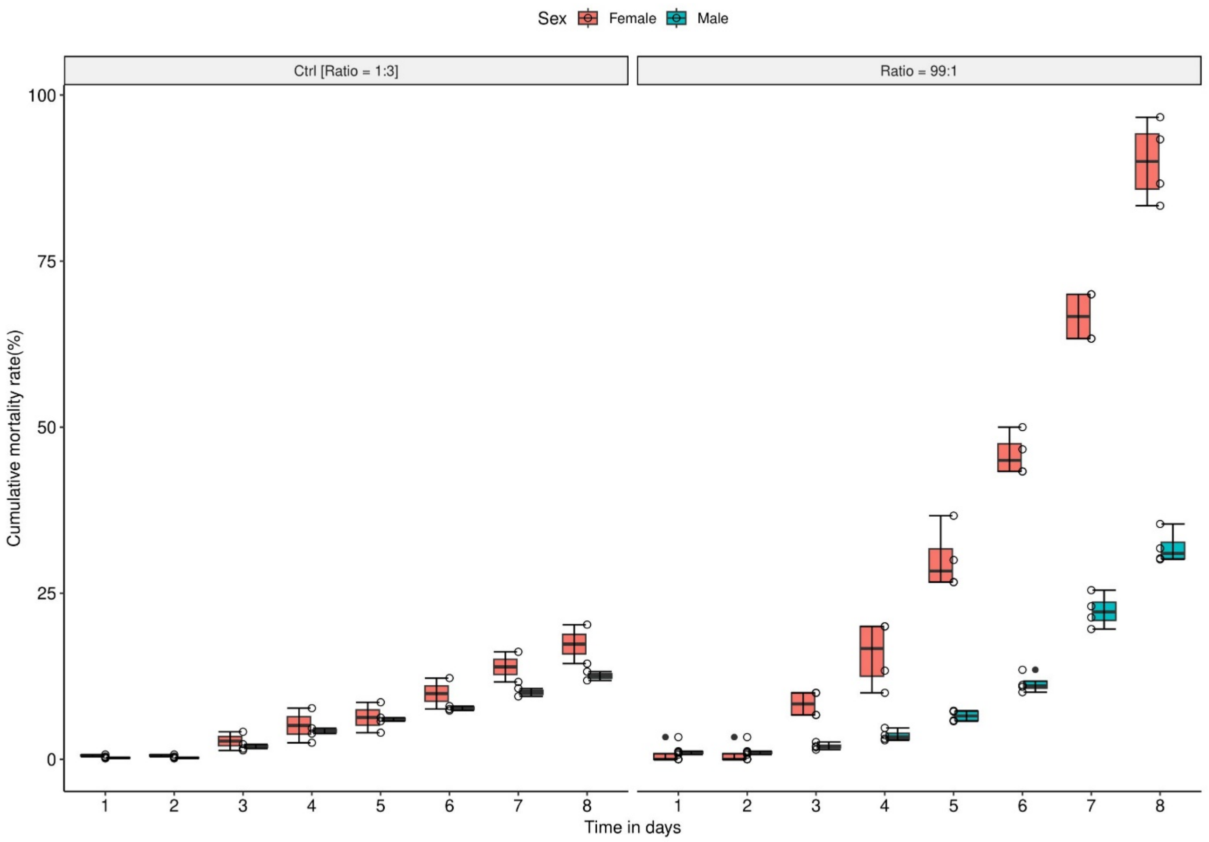

Supplementary Figure 4. Cumulative mortality rate of irradiated *Aedes albopictus* exposed to two sex ratios over 8 days. The box plots present median values and quartiles, whiskers the 95% percentiles and dots the individual data points. One-tailed pairwise multiple comparisons were performed (P value adjustment with Tukey method) using the function *emmeans ()* of the emmeans package to investigate the significance of the increase in cumulative mortality rate at different sex ratios as compared to the control (Ctrl). Mortality of females reached 90% (SD=6.1) at 8 days for a ratio of 99:1 as compared to 17.3% (SD=4.1) in the control group (odds ratio = 0.158, SE = 0.016, *z.ratio* = -17.575, $P < 10^{-4}$). The number of biologically independent replicates was n =2 for the sex ratio 1:3 (control) and n = 4 for ratio 99:1. Source data are provided in the Source Data file named “raw_data_lab&semi-field.xlsx”.

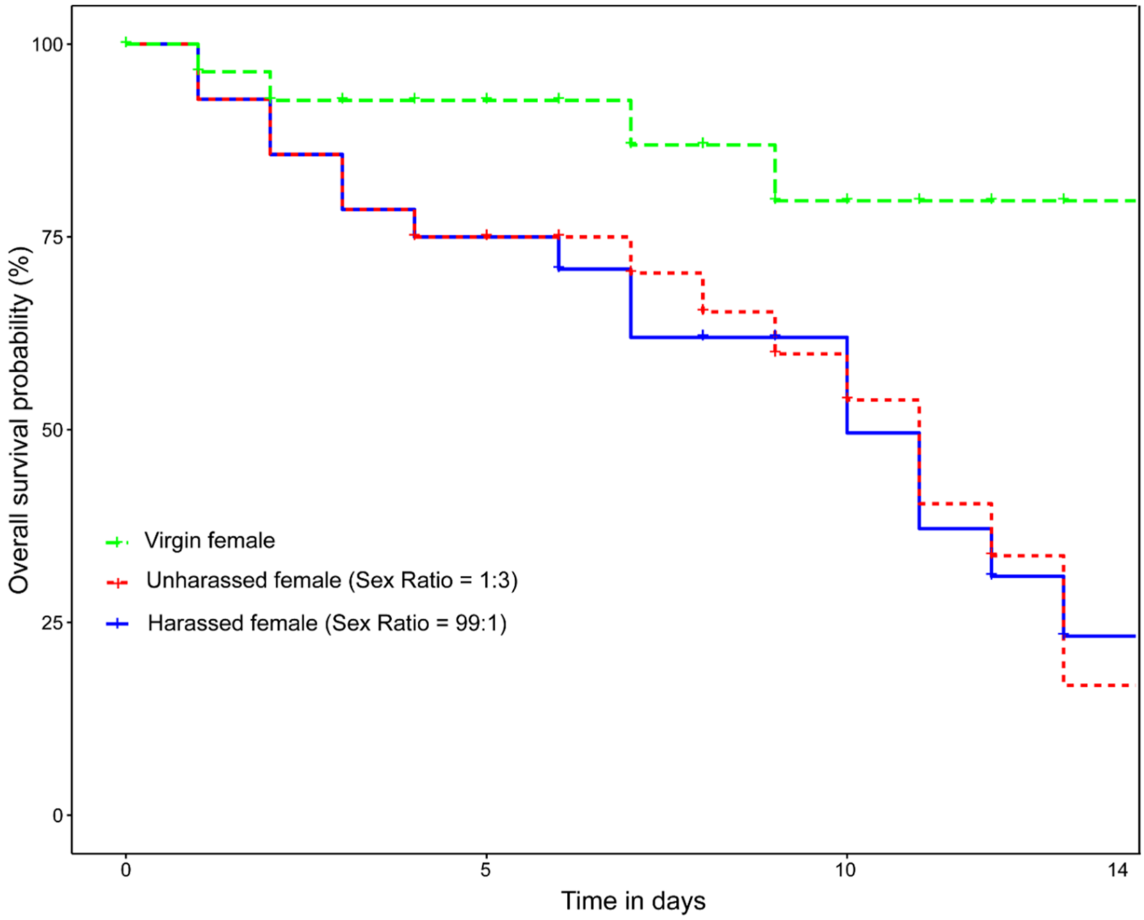

Supplementary Figure 5. Survival curves of female *Aedes aegypti* exposed to three treatments (harassed female, unharassed female and virgin female) over 14 days. Multiple Comparisons of Survival Curves are based on the function pairwise_survdiff of the package (survminer). No difference of survival was observed between females previously exposed to males at a 1:3 or 99:1 ratio after their separation from the males (P = 1.0000). However, females previously exposed to males at any ratio was lower than that of virgin females (P = 0.0095). The number of biologically independent replicates was n = 2 for each treatment. Source Data are provided in the Source Data file named “raw_data_lab&semi-field.xlsx”.

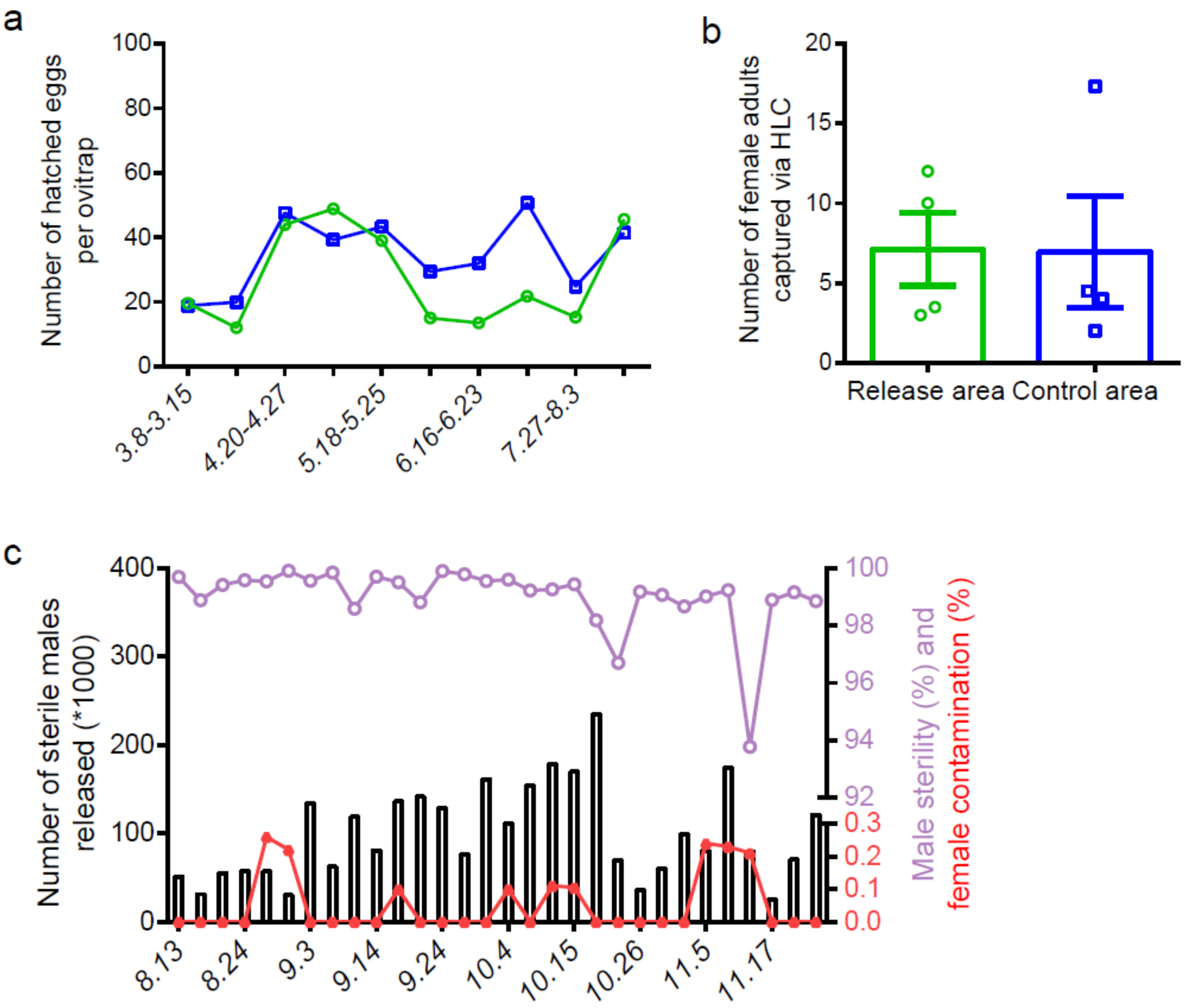

Supplementary Figure 6. Mosquito population and the release of sterile males. a, Weekly number of hatched eggs per ovitrap in the release (green dashed lines) and control area (blue dashed lines) before release. A total of 17 ovitraps were used for monitoring in the release area and 40 in the control area. No significant difference was observed in the number of hatched eggs between release and control area (n = 10 samples, t = 2.033, df = 9, P=0.0726, Two-tailed Paired t test). b, Number of female adults captured via HLC in the release (green histogram) and control area (blue histogram) before release. Two positions were selected to perform HLC in the release area and 6 positions in the control area. Four independent HLCs were performed. No significant difference was observed in the captured female adults via HLC (n = 4 samples, t = 0.0696, df = 3, P = 0.9489, Two-tailed Paired t test). c, Sterile male mosquitoes were released twice per week for a total of about 3 million males. The average female contamination rate (red dashed lines) was 0.053% (n = 30 samples, 95% CI: 0.019%-0.086%) and the male sterility (purple dashed lines) 99.03% (n = 30 samples, 95% CI: 98.59%-99.47%).

Supplementary Tables

Supplementary Table 1. Effects of various sex-ratios on the cumulative mortality rates of non-irradiated *Aedes* mosquitoes based on a mixed effects binomial linear model fit by maximum likelihood. The reference level was the Sex Ratio = 1:3 [Control]. Source data are provided in the Source Data file named "raw_data_lab&semi-field.xlsx"

Aedes species	Sex		Estimate	Std. Error	z value	Pr(>|z|)
Aedes aegypti	Female	(Intercept)	4.62	0.24	19.54	< 2e-16
		Sex Ratio = 3:7	-0.59	0.26	-2.31	0.0211
		Sex Ratio = 10:1	-0.95	0.24	-3.91	9.25e-05
		Sex Ratio = 23:2	-1.25	0.23	-5.36	8.28e-08
		Sex Ratio = 49:1	-1.13	0.24	-4.78	1.74e-06
		Sex Ratio = 99:1	-1.79	0.22	-8.03	9.92e-16
	Male	(Intercept)	4.30	0.18	24.19	<2e-16
		Sex Ratio = 3:7	0.38	0.28	1.36	0.17
		Sex Ratio = 10:1	0.53	0.29	1.81	0.07
		Sex Ratio = 23:2	0.17	0.26	0.65	0.51
		Sex Ratio = 49:1	0.58	0.30	1.96	0.05
		Sex Ratio = 99:1	0.43	0.28	1.51	0.13
Aedes albopictus	Female	(Intercept)	4.85	0.47	10.39	< 2e-16
		Sex Ratio = 50:1	-2.62	0.46	-5.67	1.47e-08
		Sex Ratio = 100:1	-2.91	0.46	-6.34	2.32e-10
	Male	(Intercept)	4.89	0.41	11.92	< 2e-16
		Sex Ratio = 50:1	-2.26	0.42	-5.36	8.42e-08
		Sex Ratio = 100:1	-2.16	0.42	-5.17	2.36e-07

Supplementary Table 2. Effects of various sex-ratios on the cumulative mortality rates of irradiated *Aedes* mosquitoes based on a mixed effects binomial linear model fit by maximum likelihood. The reference level was the Sex Ratio = 1:3 [Control]. Source data are provided in the Source Data file named "raw_data_lab&semi-field.xlsx"

Aedes species	Sex		Estimate	Std. Error	z value	Pr(>|z|)
Aedes aegypti	Female	(Intercept)	4.09	0.18	22.93	< 2e-16
	Female	Sex Ratio = 49:1	-0.61	0.19	-3.29	0.001
	Female	Sex Ratio = 99:1	-1.96	0.15	-13.13	< 2e-16
	Male	(Intercept)	3.88	0.15	26.33	< 2e-16
	Male	Sex Ratio = 49:1	1.01	0.27	3.77	0.00016
	Male	Sex Ratio = 99:1	0.27	0.17	1.58	0.115
Aedes albopictus	Female	(Intercept)	4.83	0.47	10.30	< 2e-16
	Female	Sex Ratio = 50:1	-2.24	0.46	-4.83	1.40e-06
	Female	Sex Ratio = 100:1	-2.69	0.46	-5.83	5.47e-09
	Male	(Intercept)	4.86	0.42	11.67	< 2e-16
	Male	Sex Ratio = 50:1	-2.37	0.42	-5.62	1.91e-08
	Male	Sex Ratio = 100:1	-2.50	0.42	-5.96	2.51e-09

Supplementary References

1 Guissou, E. *et al.* Effect of irradiation on the survival and susceptibility of female *Anopheles arabiensis* to natural isolates of *Plasmodium falciparum*. *Parasites & Vectors* 13, 1-11 (2020).

2 Lutrat, C. *et al.* Sex sorting for pest control: it's raining men! *Trends Parasitol.* 35, 649-662 (2019).

3 Papathanos, P. A. *et al.* Sex separation strategies: past experience and new approaches. *Malar J.* 8(Suppl 2):S5, doi:10.1186/1475-2875-8-S2-S5 (2009).

4 Lutrat, C. *et al.* Combining two Genetic Sexing Strains allows sorting of non-transgenic males for *Aedes* genetic control. *Communications Biology* 6, 646, doi:10.1038/s42003-023-05030-7 (2023).

5 WHO & IAEA. Guidance Framework for Testing the Sterile Insect Technique as a Vector Control Tool against Aedes-Borne Diseases, Geneva & Vienna. (2020).

6 Bouyer, J., Yamada, H., Pereira, R., Bourtzis, K. & Vreysen, M. J. B. Phased Conditional Approach for Mosquito Management using the Sterile Insect Technique. *Trends Parasitol.* 36, 325-336 (2020).

7 Bouyer, J., Maiga, H. & Vreysen, M. J. B. Assessing the efficiency of Verily's automated process for production and release of male *Wolbachia*-infected mosquitoes. *Nat. Biotechnol.*, 1-2 (2022).

8 Crawford, J. E. *et al.* Efficient production of male *Wolbachia*-infected *Aedes aegypti* mosquitoes enables large-scale suppression of wild populations. *Nat. Biotechnol.* 38, 482-492 (2020).

9 Oliva, C. F., Jacquet , M., Gilles, J., Lemperiere, G. & Maquart, P.-O., et al. The Sterile Insect Technique for Controlling Populations of *Aedes albopictus* (Diptera: Culicidae) on Reunion Island: Mating Vigour of Sterilized Males. *PLoS ONE 7(11): e49414. doi:10.1371/journal.pone.0049414*, doi:10.1371/journal.pone.0049414 (2012).

10 Iyaloo, D. P., Oliva, C., Facknath, S. & Bheecarry, A. A field cage study of the optimal age for release of radio-sterilized Aedes albopictus mosquitoes in a sterile insect technique program. *Entomol Exp Appl* 168, 137-147, doi:https://doi.org/10.1111/eea.12849 (2020).
