# Supplementary material for: Mating harassment may boost the effectiveness of the sterile insect technique for Aedes mosquitoes
Source: Nat Commun. 2024 Mar 4;15:1980. doi: 10.1038/s41467-024-46268-x (PMC10912119; doi:10.1038/s41467-024-46268-x)
Supplement: Supplementary file 3 — Description of Additional Supplementary Files [file 41467_2024_46268_MOESM3_ESM.pdf]

Supplementary Movie 1. Feeding inhibition of female *Aedes aegypti* through mating harassment by males. The video presents how 200 males can prevent 2 females from feeding on sugar through mating harassment.

Supplementary Movie 2. Mating behaviour of one immobilized female *Aedes aegypti* exposed to 100 males. The video shows that the female accepts 2-3 mating attempts and then reject the males. In addition, males are attracted by wing beats of the female when it tries to fly.

Supplementary Movie 3. Blood feeding inhibition of female *Aedes aegypti* in a semi-field trial at the FAO-IAEA Insect Pest Control Laboratory. The video shows how the sterile males swarm around an artificial host and reduce female feeding success through mating harassment.
