# Supplementary material for: Mating harassment may boost the effectiveness of the sterile insect technique for Aedes mosquitoes
Source: Nat Commun. 2024 Mar 4;15:1980. doi: 10.1038/s41467-024-46268-x (PMC10912119; doi:10.1038/s41467-024-46268-x)
Supplement: Supplementary file 7 — Reporting Summary [file 41467_2024_46268_MOESM7_ESM.pdf]

nature portfolio

Corresponding author(s): Jérémy Bouyer

Last updated by author(s): Feb 9, 2024

Reporting Summary

Nature Portfolio wishes to improve the reproducibility of the work that we publish. This form provides structure for consistency and transparency in reporting. For further information on Nature Portfolio policies, see our Editorial Policies and the Editorial Policy Checklist.

Statistics

For all statistical analyses, confirm that the following items are present in the figure legend, table legend, main text, or Methods section.

n/a	Confirmed	
☐	☒	The exact sample size (*n*) for each experimental group/condition, given as a discrete number and unit of measurement
☒	☐	A statement on whether measurements were taken from distinct samples or whether the same sample was measured repeatedly
☐	☒	The statistical test(s) used AND whether they are one- or two-sided<br>*Only common tests should be described solely by name; describe more complex techniques in the Methods section.*
☒	☐	A description of all covariates tested
☐	☒	A description of any assumptions or corrections, such as tests of normality and adjustment for multiple comparisons
☐	☒	A full description of the statistical parameters including central tendency (e.g. means) or other basic estimates (e.g. regression coefficient) AND variation (e.g. standard deviation) or associated estimates of uncertainty (e.g. confidence intervals)
☐	☒	For null hypothesis testing, the test statistic (e.g. F, t, r) with confidence intervals, effect sizes, degrees of freedom and P value noted<br>*Give P values as exact values whenever suitable.*
☒	☐	For Bayesian analysis, information on the choice of priors and Markov chain Monte Carlo settings
☒	☐	For hierarchical and complex designs, identification of the appropriate level for tests and full reporting of outcomes
☒	☐	Estimates of effect sizes (e.g. Cohen's d, Pearson's r), indicating how they were calculated

Our web collection on statistics for biologists contains articles on many of the points above.

Software and code

Policy information about availability of computer code

Data collection	Excel 2016
Data analysis	R version 4.2.1 (https://cran.r-project.org) using RStudio 2022.07.1 (RStudio, Inc. Boston, MA, United States, 2016), R packages lme4, ggpubr, survival, survminer, emmeans

For manuscripts utilizing custom algorithms or software that are central to the research but not yet described in published literature, software must be made available to editors and reviewers. We strongly encourage code deposition in a community repository (e.g. GitHub). See the Nature Portfolio guidelines for submitting code & software for further information.

Data

Policy information about availability of data

All manuscripts must include a data availability statement. This statement should provide the following information, where applicable:

- Accession codes, unique identifiers, or web links for publicly available datasets
- A description of any restrictions on data availability
- For clinical datasets or third party data, please ensure that the statement adheres to our policy

The raw data generated by this study and used as source data of all Figures and Supplementary Figures are provided as a Source Data file that isAll raw data are publically available (CC BY 4.0, doi:10.5061/dryad.n8pk0p31w).

Research involving human participants, their data, or biological material

Policy information about studies with human participants or human data. See also policy information about sex, gender (identity/presentation), and sexual orientation and race, ethnicity and racism.

Reporting on sex and gender	All participants were males.
Reporting on race, ethnicity, or other socially relevant groupings	For the study on human landing catch in large cages, the sample population consisted of male employees aged 18 years and above from Guangzhou Wolbaki Biotech CO., Ltd. The participants were selected based on their informed consent from the workforce of the company, excluding any individuals from ethnic minority groups or affiliated with factional organizations. All participants resided within the administrative boundaries of Guangzhou City, China.
Population characteristics	See above.
Recruitment	We first informed potential participants of our experimental objectives, protocols and requirements. They chose whether to participate or not in the experiment based on a full understanding of potential risks.
Ethics oversight	Ethics Committee on Laboratory Animal Care of the Zhongshan School of Medicine, Sun Yat-sen University

Note that full information on the approval of the study protocol must also be provided in the manuscript.

Field-specific reporting

Please select the one below that is the best fit for your research. If you are not sure, read the appropriate sections before making your selection.

☐ Life sciences ☐ Behavioural & social sciences ☒ Ecological, evolutionary & environmental sciences

For a reference copy of the document with all sections, see nature.com/documents/nr-reporting-summary-flat.pdf

Ecological, evolutionary & environmental sciences study design

All studies must disclose on these points even when the disclosure is negative.

Study description	Combined with the studies from laboratory and semi-field trials, the field trial was performed to investigate the impacts of mating harassment caused by Aedes male mosquitoes on the wild population in real conditions. We showed that high male/female ratio, e.g. >30, can result in the reduction of female longevity and female blood-feeding success, two important factors for the transmission of mosquito-borne diseases.
Research sample	This study was focused on Aedes albopictus and Ae. aegypti, two important disease vectors in the world.<br>At the IPCL, the strain of Ae. aegypti and Ae. albopictus originated respectively from Juazeiro, Brazil in 2012 (provided by Biofabrica Moscamed, IAEA Collaborative Center) and Rimini, Italy in 2018 (provided by Centro Agricoltura Ambiente, IAEA Collaborative Center). The strain of Ae. aegypti used for filming originated from Singapore and reared at the National Environment Agency–Environmental Health Institute (NEA-EHI) Singapore, mosquito production facility. At IRD, Saint-Denis, Reunion Island, Ae. albopictus originated from the island. At SYSU and JNU, Ae. albopictus originated from Guangzhou, China.
Sampling strategy	No sample-size calculations were performed. Sample size was determined to be adequate based on the magnitude and consistency of measurable differences between groups, using this guideline as a reference for the field trial: Bouyer, J. & Mamai, W. Guidelines for Mark-Release-Recapture Procedures of Aedes Mosquitoes. Vol. v 2.0 (FAO/IAEA, 2023). To measure the impact of mating harassment on female and male survival, we used sample sizes generally used at the FAO-IAEA Insect Pest Control Laboratory and showing a good statistical power (eg. Mamai, W. et al. Black soldier fly (Hermetia illucens) larvae powder as a larval diet ingredient for mass-rearing Aedes mosquitoes. Parasite 26, 57 (2019).).
Data collection	The databases for data collection were designed and documented before initiation of the experiments and collected data from each experiment were filled in excel spreadsheets within the week after data collection by the technicians in charge of running the experiments.
Timing and spatial scale	The study site is located at the North Campus of Sun Yat-Sen University in Yuexiu District, Guangzhou, China (Latitude: 23°7′39.74″N, Longitude: 113°17′22.07″E), covering an area of about 20.9 ha. Data of the field study were obtained from 2021.3-2021.12. Ae. albopictus populations were monitored before release using ovitraps collected every two weeks from 8th March to 17th August 2021. In addition, Human Landing Catch (HLC) was also performed once per month. During the release of the sterile males from mid-August to end of November 2021, ovitraps and BG traps were monitored weekly. HLC was performed three times 11 weeks post release.
Data exclusions	No data exclusions.
Reproducibility	For all lab experiments, we did many biological repeats for very similar experiments (see fig. 1, supplementary figures 1 to 4) with different mosquito strains and in two sites and always obtained a strong reduction of female survival with strong sex ratios, so there is no doubt on the reproductibility of this result. For the semi-field experiments, we did up to 12 independant biological replicared for the artificial host but only three for human and mice baits because of ethical concerns. We did not observe any failed replicate. For the lab study, we followed FAO-IAEA recommendations for sampling intensity (see corresponding section).

Randomization	Samples were allocated randomly into both experimental and control groups.
Blinding	Blinding was not possible as experimental and control sites were known by investigators. In the field study, the investigators knew the treated area because they were releasing the steril males. In the laboratory study, the investigators could observe the sex ratios when counting the dead mosquitoes.

Did the study involve field work? ☒ Yes ☐ No

Field work, collection and transport

Field conditions	The average temperature in the study area was 24.6°C in 2021 and the annual precipitation was 1,511.4 mm with a rainy season between May and October. Longitudinal series of average temperature and precipation are presented in fig. 3.
Location	The field study site was located at the North Campus of Sun Yat-Sen University in Yuexiu District, Guangzhou, China (Latitude: 23°7′39.74″N, Longitude: 113°17′22.07″E), covering an area of about 20.9 ha. The west and south areas of the campus were selected as the control area (6.55 ha), the northeast was the release area (1.17 ha), and a buffer zone (4.87 ha) was set between the release and the control area.
Access & import/export	The field site was located in a bustling metropolitan area with parks, hospitals, and residential areas nearby. There was no strict entry permits. Aedes albopictus is considered a health pest and is not classified as a protected animal in China. Therefore, experimental research can be conducted in compliance with biosafety regulations.
Disturbance	In the release site, the presence of very high number of males flying around people after release may cause concern to some people including staff conducting the experiment, although the sterile male mosquitoes do not bite. Community education was used to inform personnel of the research intention to minimise their concerns and associated disturbance by the sterile males.

Reporting for specific materials, systems and methods

We require information from authors about some types of materials, experimental systems and methods used in many studies. Here, indicate whether each material, system or method listed is relevant to your study. If you are not sure if a list item applies to your research, read the appropriate section before selecting a response.

Materials & experimental systems

n/a	Involved in the study	
☒	☐	Antibodies
☒	☐	Eukaryotic cell lines
☒	☐	Palaeontology and archaeology
☐	☒	Animals and other organisms
☒	☐	Clinical data
☒	☐	Dual use research of concern
☒	☐	Plants

Methods

n/a	Involved in the study	
☒	☐	ChIP-seq
☒	☐	Flow cytometry
☒	☐	MRI-based neuroimaging

Animals and other research organisms

Policy information about studies involving animals; ARRIVE guidelines recommended for reporting animal research, and Sex and Gender in Research

Laboratory animals	Mosquitoes (Aedes albopictus and Aedes aegypti), Mice
Wild animals	Wild mosquitoes (wild Aedes albopictus strain with wAlbA and wAlbB double infections) were sampled with traps and by human landing catches. They were killed by freezing immediately after capture by putting the capture cages in ice boxes containing ice, which were also used to transport them to the laboratory, located less than 500m apart. The age of the specimen was unknown. Wild eggs collected from the field (same species and strain) were transported and incubated in the insectarium at ambiant temberature.
Reporting on sex	Sex was fully integrated in the analysis since our study is on the impact of sex-ratio on female survival, feeding success and catches.
Field-collected samples	Each captured adult mosquito was immediately killed and stored separately in a 1.5 mL tube maintained at -20°C before Wolbachia detection by PCR. There was no maintenance of live specimens in the laboratory. All the experimental mosquitoes were kept at -20°C for more than 16h before being discarded to ensure biosafety. Eggs collected from the field and hatch larvae were boiled before being discarded to ensure biosafety.
Ethics oversight	This research complies with all relevant ethical regulations. For the study involving Human Landing Catch in large cages, the protocol was approved by the Ethics Committee on Laboratory Animal Care of the Zhongshan School of Medicine (ZSSOM), Sun Yat-sen University (No. 2018-020). The experiment involving the use of anesthetized mice to blood-feed Aedes mosquitoes was conducted

according to protocols on Laboratory Animal Care approved by ZSSOM (03/14-036-00 and No. 2017-041). The field trial on applying SIT for Aedes albopictus control has also been reported to and approved by ZSSOM before the release of sterile males in 2021.

Note that full information on the approval of the study protocol must also be provided in the manuscript.
